# Puffed Up Creativity? The Relationship Between Grandiose Narcissism and Creativity: A Study of Chinese High-Tech Companies

**DOI:** 10.3390/bs15030273

**Published:** 2025-02-26

**Authors:** Wen Zheng, Huihui Yu, Yu Gu, Yang Huang

**Affiliations:** 1 Department of Medical Psychology, Capital Medical University, Beijing 100069, China; 2 School of Psychological and Cognitive Sciences, Peking University, Beijing 100871, China; huihuiyu@stu.pku.edu.cn; 3 Department of Epidemiology and Health Statistics, School of Public Health, Capital Medical University, Beijing 100069, China; guyu2004@mail.ccmu.edu.cn; 4 Department of Psychology, Tsinghua University, Beijing 100084, China; huangyang19@tsinghua.edu.cn

**Keywords:** creativity, grandiose narcissism, vulnerable narcissism, self-esteem

## Abstract

Prior research has yielded inconsistent findings regarding the association between narcissism and creativity, possibly due to the neglect of different narcissism subtypes. The present study aimed to elucidate the relationship between narcissism and creativity by introducing two distinct subtypes of narcissism, namely grandiose and vulnerable narcissism. A total of 571 employees (267 males, 304 females) from high-tech enterprises across 26 provinces in China completed measures of narcissism, creative performance, creative states, and self-esteem, following explicit instructions. The results indicated that participants with grandiose narcissism reported significantly higher levels of creativity states and fluency in creativity performance than those with vulnerable narcissism. Self-esteem mediated the relationship between both subtypes of narcissism and creativity. The implications of the revealed differential effects of grandiosity and vulnerability on creativity in the workplace were also discussed.

## 1. Introduction

Self-appreciation, a core principle of modern American culture, has gradually influenced global culture through the process of globalization. While self-appreciation can enhance one’s well-being and life satisfaction, excessive self-appreciation can lead to a detrimental consequence: narcissism. Narcissists are characterized by a sense of self-importance, entitlement, and superiority, and they are often found among talented artists. The relationship between narcissism and creativity has attracted the attention of psychologists (Furnham et al., 2013; Goncalo et al., 2010; Raskin, 1980), who have explored the correlation between narcissism and various aspects of creative personality, such as instability, ambition, and originality. Previous studies have shown that narcissistic people tend to engage in creative activities (Martinsen et al., 2019) and exaggerate their performance, resulting in higher score in self-rated creativity tasks (Goncalo et al., 2010; Jonason et al., 2017). However, other researchers have argued that narcissism has no association with creativity (Goncalo et al., 2010). The inconsistency in the findings may stem from the different definitions and conceptualizations of narcissism, which can vary in their meaning and implications. (Rohmann et al., 2012). Wink (1991) identified two subtypes of narcissism: grandiose (or overt) narcissism and vulnerable (or covert) narcissism. These subtypes may differ in their creativity outcomes due to their distinct orientations and motivations. Previous research typically regard narcissism as a holistic trait, without considering the differences in creativity across subtypes of narcissism, resulting in contradictory findings. The current study aims to examine the relation between narcissism and creativity from the perspective of different subtypes of narcissism, to gain a deeper understanding of this complex phenomenon.

### 1.1. Narcissism

The definition of narcissism has historically varied in the various branches of psychiatry and psychology (Ackerman et al., 2019). Generally, narcissism is characterized by arrogance, a sense of entitlement, and a belief that one is special and unique, and it is often accompanied by arrogant behavior and a lack of empathy (DSM-IV; American Psychiatric Association, 2000). However, a growing consensus among researchers is that narcissism can be divided into two main subtypes: grandiose and vulnerable (e.g., Wink, 1991; Miller et al., 2011; Pincus et al., 2009). Both subtypes share some core features of narcissism, including entitlement, disregard for others, need for recognition, and grandiose fantasies (Maciantowicz et al., 2022). Nevertheless, grandiose and vulnerable also differ in significant ways. For instance, grandiose narcissism is characterized by a desire to maintain a false self-image, a tendency to display oneself, and a strong need for affirmation from others, whereas vulnerable narcissism is associated with preoccupation with grandiose fantasies, oscillation between feelings of superiority and inferiority, and fragile self-confidence (Rohmann et al., 2012). Although most experts emphasized the grandiosity over the vulnerability (Cain et al., 2008), recent work has begun to conceptualize and assess narcissistic states corresponding to grandiosity and vulnerability. Grandiose narcissism aligns with the social–personality framework of narcissism, whereas vulnerable narcissism corresponds more closely to its clinical conceptualization (Miller & Campbell, 2008).

Samuel and Widiger (2008) investigated the relationships among five self-report measures of narcissism and narcissistic personality disorder (NPD), revealing notable differences in how these measures correlated with extraversion (rs ranging from 0.15 to 0.48) and neuroticism (rs ranging from 0.40 to 0.13). Notably, the personality profile associated with the vulnerable narcissism factor showed a negative yet non-significant correlation with the profile linked to grandiose narcissism. Consistent with previous research (Miller & Campbell, 2008; Miller et al., 2010), the two forms of narcissism exhibit substantial divergence, particularly in their associations with neuroticism and extraversion, and also show significant differences in global self-esteem (Miller et al., 2011).

Previous studies on the relationship between narcissism and creativity have rarely explored the different subtypes of narcissism in relation to creativity, and it is possible that the different psychological and behavioral characteristics of grandiose and vulnerable narcissism show very different patterns of relationship in relation to creativity. This may account for the contradictory results that have emerged in the past regarding the relationship between narcissism and creativity.

### 1.2. Narcissism and Creativity

To investigate the relationship between narcissism and creativity, it is necessary to initially identify which aspects of creativity to examine. Early scholars in this area, such as Kris (1952), and later Kohut (1966), mainly described narcissism as fostering artistic playfulness, suggesting that narcissism could be related to those personality traits that motivate creative self-expression. Recent psychological studies expanded the understanding of narcissism beyond pathological disorders, considering it a trait linked to self-appreciation, egocentric exceptionalism, and social selfishness (Sedikides, 2021).

However, previous findings on the relationship between narcissism and creativity have yielded inconsistent results. Some studies suggest that narcissistic individuals exhibit higher self-perceived creativity, with narcissism positively correlated with self-rated creative ability (Furnham et al., 2013; Goncalo et al., 2010; Raskin, 1980). Recent meta-analytic findings further confirm that narcissism is positively associated with self-reported creativity but not necessarily with objective creative performance (Lebuda et al., 2021). It seems that narcissistic people are more prone to overestimate their creativity relative to others and to show more self-enhancement when judging their own performance.

Moreover, Ji et al. (2023) demonstrated that the relationship between narcissism and creativity is strongly influenced by social perception—narcissists appear more creative mainly because others believe them to be. This aligns with findings from Chang and Gong (2023), who proposed a serial mediation model in which narcissism enhances creative self-efficacy, which in turn promotes actual creative behavior, but only when external validation is present. These results indicate that narcissists’ creativity may be driven more by their motivation to impress others rather than by an inherent creative advantage.

Additionally, studies suggest that different facets of creativity may be influenced by narcissism in distinct ways. Narcissistic individuals tend to generate more ideas (fluency) but do not necessarily produce more original or flexible solutions (Chang & Gong, 2023; Martinsen et al., 2019). Furthermore, narcissism appears to be more strongly linked to radical creativity—highly novel but often impractical ideas—rather than incremental creativity, which focuses on refining and improving existing concepts.

One potential reason for the inconsistent findings across studies of the relationship between narcissism and creativity is the existence of different subtypes of narcissism. While most research has focused on grandiose narcissism, which is typically associated with confidence, dominance, and self-admiration, vulnerable narcissism presents a contrasting profile characterized by insecurity and hypersensitivity (Dickinson & Pincus, 2003). Recent evidence suggests the positive link between narcissism and creativity is more pronounced in individuals with high grandiose narcissism, while those with highly vulnerable narcissism may experience more self-doubt, limiting their creative engagement (Chang & Gong, 2023). This distinction could account for some of the contradictory results found in past research.

### 1.3. Self-Esteem as a Mediator

Substantial amounts of research have demonstrated a high correlation between narcissism and self-esteem (Bosson et al., 2008; Brummelman et al., 2016; Campbell et al., 2002; Zeigler-Hill & Besser, 2013). Socially, those with high self-esteem are as confident and outgoing as narcissists (Gebauer & Sedikides, 2018). Moreover, the positive social feedback associated with narcissism helps to maintain high self-esteem. Narcissism enhances and develops the ideal self in an exaggerated manner to regulate self-esteem. Both concepts involve a positive self-image; thus, a positive correlation seems evident (Bosson et al., 2008; Sedikides et al., 2004), and the statistics clearly supported this (Ackerman et al., 2011; Orth et al., 2016).

However, when narcissism is divided into different subtypes, grandiose and vulnerable narcissism have markedly different correlations with self-esteem (Miller et al., 2011). Grandiose narcissism tends to constantly seek praise and associate themselves with highly positive and successful others to boost their power, status, and self-esteem (Zeigler-Hill & Besser, 2013), whereas vulnerable narcissism involves expressions of low self-esteem, defensiveness, and weakness (Weiss & Miller, 2018; Hart et al., 2020). Vulnerable narcissism exhibits feelings of inadequacy, inferiority, and worthlessness that do not appear to match with high self-esteem (Morf & Rhodewalt, 2001). The divergent relationships between these forms of narcissism and self-esteem indicate fundamental differences in these subtypes (Bosson et al., 2008).

Silvia and Phillips (2004) discovered that people with higher self-esteem scored higher on creativity. Those with higher self-esteem were less afraid of setbacks, and performed better on creative tests (Kemple et al., 1996). Deng and Zhang (2011) conducted a meta-analysis of 24 studies on the relationship between self-esteem and creativity and found that the correlation coefficient between self-esteem and creativity was stable and significantly positive.

### 1.4. The Present Study

Previous studies have yielded inconsistent results regarding the association between narcissism and creativity, possibly due to insufficient consideration of different subtypes of narcissism. To clarify the relationship between narcissism and creativity, the current study introduced two narcissistic subtypes (i.e., grandiose and vulnerable narcissism). All participants completed scales measuring their narcissism, creative performance, creative states and self-esteem. Moreover, to better understand the psychological mechanisms underlying the creative differences, this study investigated the mediating role of self-esteem in the relationship between narcissism and creativity.

Given the nature of narcissism, creativity and self-esteem, we proposed the following hypotheses: (1) participants with grandiose narcissism would have significantly higher levels of self-rated creativity than those with vulnerable narcissism; (2) participants with grandiose narcissism would report significantly higher levels of fluency in other-rated creativity than those with vulnerable narcissism; and (3) self-esteem would mediate the relationship between subjects with different narcissistic subtypes and their self-rated creativity states and other-rated creativity performance.

## 2. Method

### 2.1. Participants

A total of 571 employees (267 males, 304 females) from high-tech enterprises across 26 provinces in China participated in the study. Their mean age was 32.3 years (SD = 6.9) with a range of 17 to 58. To ensure a representative sample from the high-tech sector, we established collaboration with HR departments of multiple enterprises, who assisted in study invitations and participant outreach. All participants were recruited through a widely used Chinese social network app, WeChat, via targeted advertisement specially aimed at professionals in high-tech sector. Participants who completed the study were provided with a monetary compensation (about USD 3) as an incentive. This sample provides a unique opportunity to explore creativity and personality traits of China’s rapidly growing high-tech industry.

The study followed the guidelines of Human Research Ethics Committees of Capital Medical University (grant number: Z2023SY047). To ensure confidentiality, participants were informed that their responses would remain anonymous, and only aggregated data would be used for analysis. All participants provided written informed consent electronically through an encrypted survey platform, ensuring compliance with ethical standards. We affirm that such consent was in accordance with the Declaration of Helsinki.

### 2.2. Procedure

Participants were informed of the nature of the study, gave consent and completed all the measures mentioned above. Participants reported their current creativity state, and then completed the alternative uses task, using a brick and a paper box as stimuli. The order of the tasks was counterbalanced, such that half of them completed the brick task first, while the other half of them completed the box task first. Then they responded to the trait measurements. They reported demographics and were thanked and debriefed upon completion. All data were collected online.

This study employed a cross-sectional survey design to examine the relationship between narcissism subtypes and creativity. All data were collected at a single time point, and no experimental manipulation was conducted, reflecting associational patterns rather than causal effects between narcissism and creativity.

### 2.3. Measures

The Brief Pathological Narcissism Inventory (B-PNI, Schoenleber et al., 2015) was used to measure narcissistic grandiosity and narcissistic vulnerability. It was developed from the Pathological Narcissism Inventory (PNI; Pincus et al., 2009) and contained 28 self-report items, including grandiose narcissism (e.g., “I can usually talk my way out of anything.”) and vulnerable narcissism (e.g., “When people don’t notice me, I start to feel bad about myself.”). Participants indicated their agreement to each item on a 5-point scale ranging from 1 (not at all like me) to 5 (very much like me). Items for grandiose and vulnerable narcissism were averaged to indicate grandiose narcissism (Cronbach’s *α* = 0.79) and vulnerable narcissism (Cronbach’s *α* = 0.89). The B-PNI has been previously used in studies on Chinese populations (Pincus, 2023), but cultural differences in narcissistic expression should be considered when interpreting the results.

The 10-item Rosenberg Self-Esteem Scale (RSE, Rosenberg, 1965) was used to measure participants’ global self-esteem. Half of which were phrased in a negative direction (e.g., “All in all, I am inclined to think that I am a failure.”) and half in a positive direction (e.g., “On the whole, I am satisfied with myself.”). Respondents rated their agreement extent to each item on a 5-point Likert scale ranging from 1 (disagree strongly) to 5 (agree strongly). Items were averaged to indicate self-esteem (Cronbach’s *α* = 0.78).

We measured self-reported general creativity perception with a 4-item author-constructed measure: fluency, usefulness, originality, and general creativity ability, based on Guilford’s (1967) theoretical framework and expanded by Runco and Jaeger (2012). These components provide a comprehensive understanding of individual creativity. Participants indicated their creativity on fluency, usefulness, originality and general creativity ability on 1 (not at all) to 5 (very much) Likert scales, with item like “How do you think your creativity in fluency is today”, reflecting participants’ perceptions of their creative abilities in terms of fluency, originality, and usefulness. Given that we created this measure, we ran a principal component analysis with a varimax rotation. These items loaded well on a single factor (Eigen = 3.07; 76.78% of the variance) and were averaged to create a single index of self-reported creativity (Cronbach’s *α* = 0.90).

Creative performance was measured by two alternative uses tasks (Gilhooly et al., 2007) on the line, a widely recognized and validated measure of divergent thinking chosen to ensure objective and reliable assessment of creativity, minimizing common method biases and external confounds that may arise in real-world work settings. The AUT allows for direct evaluation of creative fluency, flexibility, and originality, making it particularly suitable for assessing individual differences in creativity. Rather than assessing creativity within a specific workplace context, the AUT captures an individual’s fundamental creative potential, which serves as the cognitive foundation for creative performance across various domains (Runco & Acar, 2012). Prior research has demonstrated that divergent thinking, as measured by AUT, strongly correlates with real-world creative accomplishments and professional innovation (Benedek et al., 2014). Participants were given 5 min per task to provide unusual uses as many as possible. We assessed their creativity performance on three dimensions: fluency, flexibility, and originality. Participants collected responses across two tasks were independently scored by 3 trained raters.

Creativity in this study was assessed using both self-reported and other-rated measures, ensuring a balanced and multidimensional approach to capturing creativity.

### 2.4. Rating

A training session was adopted as opposed to more lengthy inter-rater reliability procedure to save time. A random selection of 1/5 of the data was assessed collectively on three dimensions mentioned above by the three raters and the first author, to reach consensus and to standardize evaluations (Jonason et al., 2017).

First, the three raters counted the number of unusual uses each participant offered to the two tasks. For example, with the target item “brick”, if a participant’s response was to be used to build (a wall, a house, or a building) indicated a usual use, whereas using a brick as equipment to keep fit indicated an unusual use. Each rater independently counted the number of each participant’s unusual responses to each task and arrived a rating for each participant (Cronbach’s *α*_brick_ = 0.97, Cronbach’s *α*_box_ = 0.99, across raters). The ratings of all three raters were averaged across two tasks as an index of fluency of other-rated creativity performance (Cronbach’s *α* = 0.96).

Second, the same three raters counted the numbers of different categories the responses included. For example, with the target item “box”, if a participant’s responses were to be used to contain fruits, contain toys, or contain books, indicated one category, whereas using a box as a bin, a flowerpot, or a shelter, then it concerned 3 categories. Each rater independently counted the responses each participant offered and reached a rating for each participant (Cronbach’s *α*_brick_ = 0.93, Cronbach’s *α*_box_ = 0.87, across raters). We averaged the number of the categories offered by each rater to create an index of flexibility of other-rated creativity performance (Cronbach’s *α* = 0.91).

Third, participants responses on each task were independently scored by the same raters for originality (1 = not at all, 5 = very much). For example, with the target item “brick”, if participants said it was to be used to build a house, it was scored a one, whereas, it was used to be a pillow, it was scored a five. The inter-rater reliabilities of originality on two tasks were acceptable (Cronbach’s *α*_brick_ = 0.75, Cronbach’s *α*_box_ = 0.77, across raters). The ratings from the three raters were averaged across both tasks to indicate originality of other-rated creativity performance (Cronbach’s *α* = 0.81).

The internal consistency coefficient of fluency, flexibility and originality are quite high across two tasks (Cronbach’s *α* = 0.95).

### 2.5. Data Analysis

Narcissistic subtypes. We changed grandiose narcissism and vulnerable narcissism into a Z-score. To specify either trait, we coded participants into “1” as narcissistic grandiosity when their standard deviation (SD) of narcissistic grandiosity >1 as well as that of narcissistic vulnerability <1, offering further insights and clarification in comparison to previous research. Participants with a SD of narcissistic vulnerability >1 as well as that of narcissistic grandiosity <1 into “2” as vulnerable narcissism predominates over grandiose narcissism, while an SD of narcissistic grandiosity <−1 as well as narcissistic vulnerability <−1 as the non-narcissism participants. There was no overlap between these 3 groups. Data from 110 participants will be further analyzed based on this criterion.

This classification approach, though not a standard in narcissism research, is commonly used in personality psychology to facilitate subgroup comparisons (e.g., Cohen, 1988). However, to ensure that our findings are robust beyond categorical classification, we also conducted a structural equation modeling (SEM) analysis, where narcissism was treated as a continuous variable, allowing us to directly estimate the relationships between narcissism subtypes and creativity measures via regression coefficients.

We examined missing data patterns and applied multiple imputation using the MICE package in R (4.4.2) to minimize potential biases.

## 3. Results

### 3.1. Difference Between Narcissistic Grandiosity and Narcissistic Vulnerability in Creativity

The descriptive statistics of variables were shown in Table 1.

Self-reported creativity state. The results of repeated measurement MANOVA indicated that types of narcissism had a significant main effect on self-reported creativity, *F* (2, 107) = 10.13, *p* < 0.01, *η*^2^ = 0.16. LSD indicated that the self-reports of creativity of narcissistic grandiosity were significantly higher than those of narcissistic vulnerability and non-narcissism. However, there was no significant difference between narcissistic vulnerability and non-narcissism in self-report creativity state, *p* > 0.05.

**Figure 1:**
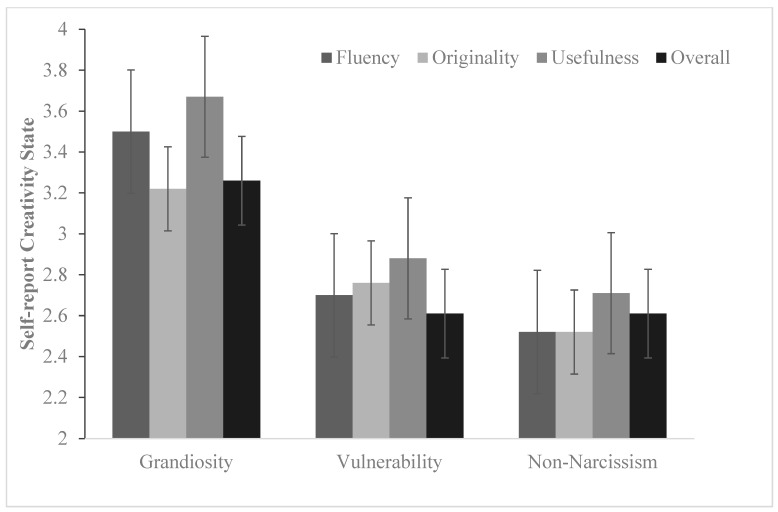
Mean differences in self-reported creativity state across narcissism subtypes (cross-sectional survey results). Note: These results reflect observed group differences within a cross-sectional dataset and should not be interpreted as evidence of causal relationships.

Significant differences were found between different facets of creativity, *F* (3, 321) = 4.67, *p* < 0.05, *η*^2^ = 0.04. LSD indicated that the self-reported originality, fluency of creativity and overall creativity state were significantly higher than usefulness of creativity, *p*_originality_ < 0.01, *p*_fluency_ < 0.05, *p*_overall_ < 0.01, shown as Figure 1.

However, types of narcissism and facets of creativity had no significant interactions, *p* > 0.05.

**Figure 2:**
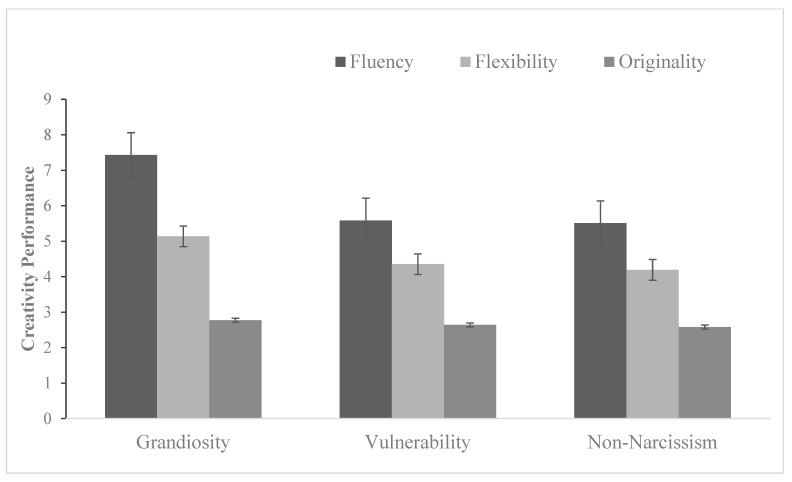
Mean differences in other-rated creativity performance across narcissism subtypes (cross-sectional survey results). Note: The data represent correlational associations rather than causal effects due to the cross-sectional nature of the study.

Other-rated creativity performance. The results of RM-MANOVA indicated that interactions of narcissism types and creativity dimensions were significant, *F* (4, 214) = 3.40, *p* < 0.05, *η*^2^ = 0.06. Further analysis indicated that narcissistic grandiose participants received greater scores in fluency, *F* (2, 109) = 3.34, *p* < 0.05, while no significant differences between narcissistic vulnerable and non-narcissistic participants in either flexibility or originality of other-rated creativity performance, *p* > 0.05, shown as Figure 2.

Types of narcissism had marginally significant main effect on other-rated creativity performance, *F* (2, 107) = 2.95, *p* = 0.056, *η*^2^ = 0.05.

Significant differences were found between different facets of creativity, *F* (2, 214) = 111.69, *p* < 0.01, *η*^2^ = 0.51. LSD indicated that fluency was significantly higher than flexibility and originality.

### 3.2. Correlations Between Narcissism and Individual Differences in Creativity

As for self-reported creativity state, grandiose narcissism was significantly positive related to originality (*r* = 0.17, *p* < 0.01), flexibility (*r* = 0.22, *p* < 0.01), usefulness (*r* = 0.19, *p* < 0.01), and overall self-reported creativity state (*r* = 0.19, *p* < 0.01).

As for other-rated creativity performance, grandiose narcissism was significantly positive related to fluency (*r* = 0.18, *p* < 0.01), flexibility (*r* = 0.18, *p* < 0.01), and originality (*r* = 0.15, *p* < 0.01).

However, the results of vulnerable narcissism did not copy that of grandiose narcissism. The relationship between vulnerable narcissism and creativity, neither the self-reported creativity state or other-rated creativity performance, was not significant, *p* > 0.05, shown as Table 2.

### 3.3. Mediation of Self-Esteem in the Relationship Between Different Subtypes of Narcissism and Creativity

**Figure 3:**
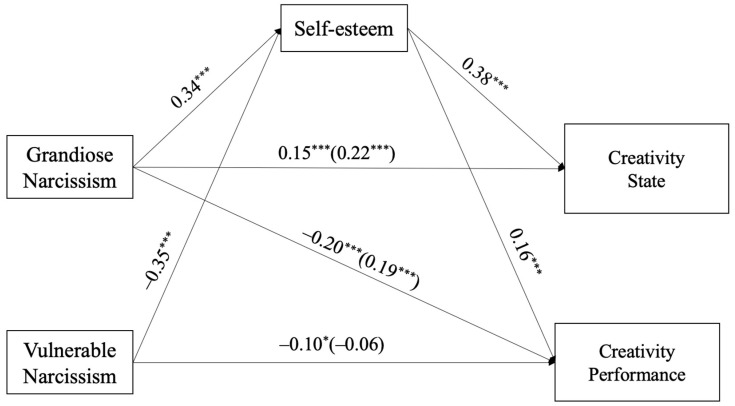
The tested path analytical model for the hypothetical mediation model. *Note*: * *p* < 0.05; *** *p* < 0.001.

Results show that grandiose narcissism positively predicts creative states (β = 0.15, *p* < 0.001) and positively predicts creativity performance (β = 0.20, *p* < 0.001). Grandiose narcissism positively predicts self-esteem (β = 0.34, *p* < 0.001). Vulnerable narcissism negatively predicts creativity performance (β = −0.10, *p* < 0.05) and negative predicts self-esteem (β = −0.35, *p* < 0.001). Meanwhile, self-esteem positively predicts creative states (β = 0.38, *p* < 0.001) and positively predicts creativity performance (β = 0.16, *p* < 0.001), shown as Figure 3.

Participants who were scored higher in grandiose narcissism were likely to report higher levels of self-esteem, and then they were inclined to report higher creative states and gain higher score in creative tasks. Those who scored higher in vulnerable narcissism were likely to receive lower scores in self-esteem and then receive lower marks in creative tasks.

To test the hypothesized indirect effect of self-esteem, we used 5000 bootstrap samples to estimate the effect size and 95% confidence intervals. Table 3 shows the standardized effect size and 95% confidence intervals of indirect, direct, and total effects, respectively. The indirect path from grandiose narcissism to creative states is significant (*R*^2^ = 0.16, 95% CI [0.08, 0.22], *p* < 0.01), indicating that self-esteem partially mediated the relationship between grandiose narcissism and creative states. The indirect path from grandiose narcissism to creative performance is significant (*R*^2^ = 0.17, 95% CI [0.12, 0.28], *p* < 0.01), indicating that self-esteem also partially mediated the relationship between grandiose narcissism and creative performance. Self-esteem plays a partial mediation role in the relationship between vulnerable narcissism and creative performance (*R*^2^ = −0.16 95% CI [−0.18, −0.02], *p* < 0.05).

## 4. Discussion

The present study examines the relationship between different subtypes of narcissism and creativity, as well as the mediating role of self-esteem in both. The results indicated that participants with grandiose narcissism reported significantly higher levels of self-reported creativity than those with vulnerable narcissism or non-narcissists. Moreover, participants with grandiose narcissism performed significantly better in fluency, one of the dimensions of other-rated creativity than vulnerable and non-narcissistic participants, while there was no significant difference between grandiose and vulnerable narcissism in flexibility and originality, the other two dimensions of other-rated creativity. Self-esteem mediated the relationship between both subtypes of narcissism and creativity.

The present study contributes to the literature by resolving the conflicting findings of previous research on the relationship between narcissism and creativity (Furnham et al., 2013; Martinsen et al., 2019; Rohmann et al., 2012). While previous studies have examined the link between narcissism and creativity broadly, they often fail to differentiate between the two major subtypes of narcissism—grandiose and vulnerable. Our study extends existing research by systematically distinguishing these two subtypes and demonstrating their distinct impacts on creativity. Unlike previous findings that generally associate narcissism with enhanced creativity (Goncalo et al., 2010), our results reveal a more nuanced pattern: grandiose narcissists report and demonstrate higher levels of fluency in creativity, whereas vulnerable narcissists do not show the same advantage. This suggests that the link between narcissism and creativity is not uniform but depends on the specific subtype of narcissism, highlighting the need for a more differentiated theoretical framework, which is supported by Mann’s (2022) study.

We also compared the association between narcissism and creativity in people with three different personality traits: grandiose narcissism, vulnerable narcissism, and non-narcissistic, and clarified the distinctive features of each group in terms of creativity. Our findings indicate that the relationship between narcissism and creativity differs substantially between grandiose and vulnerable narcissists. Grandiose narcissists’ inflated self-perception and desire for admiration drive them to engage in creative endeavors and report higher creative tendencies, even when their objective performance does not always align with their self-assessment (Jauk & Sordia, 2018; Geukes et al., 2017). This relationship can be better understood through the lens of self-enhancement theory (Sedikides & Gregg, 2008), which posits that individuals with grandiose narcissistic traits are highly motivated to maintain and enhance a superior self-image (Gregg & Sedikides, 2010). Creativity, particularly when it is recognized and rewarded, provides an opportunity for such self-enhancement. Grandiose narcissists may engage in creative tasks not only as an expression of their abilities but also to gain admiration and assert dominance in professional and social settings. Conversely, vulnerable narcissists, characterized by self-doubt and heightened sensitivity to evaluation, may exhibit creativity in more nuanced ways. While they do not report high levels of creativity, their analytical thinking and focus on originality may suggest an alternative creative pathway. However, their fear of failure and social scrutiny might prevent them from actively showcasing their creative potential. This highlights the need for a more refined theoretical perspective on how different narcissistic subtypes influence creative expression. Because of the significant disparities in personality features between grandiose and vulnerable narcissism, differently structured narcissistic populations among participants should be evaluated in future studies. Furthermore, the present study also explored the mediating role of self-esteem in the relationship between different subtypes of narcissism and creativity, to gain a deeper insight into the psychological factors that underlie the association between different forms of narcissism and creative abilities.

The findings of the present study have great significance on creativity promotion in the workplace. Given that grandiose narcissists report higher levels of self-perceived creativity, organizations can strategically leverage this confidence by assigning them roles that require idea generation and quick, high-volume output, such as marketing, product ideation, or brainstorming sessions. However, as their creativity advantage lies primarily in fluency rather than originality, it is essential to complement their contributions with team members who excel in analytical and originality-driven tasks. On the other hand, vulnerable narcissists, despite reporting lower self-perceived creativity, may still possess creative potential in areas requiring deep analysis and originality. Due to their heightened sensitivity to evaluation, organizations can foster their creativity by implementing low-pressure, collaborative settings where they feel psychologically safe to share ideas. Structured feedback mechanisms and supportive team dynamics can help unlock their creative contributions without triggering their fear of negative evaluation. Additionally, companies should consider distributing creative tasks based on these differences—assigning grandiose narcissists to roles emphasizing fluency and visible idea generation, while engaging vulnerable narcissists in projects that require in-depth research, originality, and problem-solving.

This study employed a methodological approach that incorporated both subjective reports and objective assessments to assess the level of creativity and its outcomes. As a self-reported measure, the findings primarily reflect participants’ subjective perceptions of their creativity, which may differ from objective measures or expert evaluation. Previous research has mainly relied on a single measure, such as self-reported creative states (Han et al., 2019) or performance on specific creative tasks or tests. This singular approach has limited our ability to examine various aspects of creativity holistically. In this study, we combined self-reported and other-rated measures to assess creativity, offering a multidimensional perspective on participants’ creative abilities. This dual approach addresses potential biases associated with relying solely on self-perception or other-rated evaluations. The high inter-rater reliability of the other-rated creativity scores further strengthens the validity of our findings. Nevertheless, we recognize that creativity is a complex and dynamic construct and may fluctuate across different contexts and emotion states. Future studies may benefit from integrating alternative methods, such as task-based longitudinal designs or real-time assessments (e.g., experience sampling method, ESM), to complement the current framework and capture creativity in diverse contexts.

While our study provides valuable insights, there are several limitations to consider. Firstly, the present study employed a cross-sectional design to explore the association between narcissism and creativity, without accounting for the influence of temporal (Goncalo & Krause, 2014) and situational factors (Mao et al., 2021; Sternberg & Karami, 2021; Tromp & Sternberg, 2022) in this relationship. Also, due to the cross-sectional nature of this study, we cannot establish causal relationships between narcissism, self-esteem, and creativity. Future studies may benefit from integrating temporal variables alongside additional contextual elements, adopting longitudinal or experimental designs to examine the dynamics and causal mechanisms underlying these associations. Secondly, the assessment of the dependent variable in this study primarily centered only on the various elements of divergent thinking that are indicative of creativity. While previous research has mostly emphasized the aspect of divergent thinking concerning creativity (Acar & Runco, 2019; Zhang et al., 2020), it is important to acknowledge that convergent thinking also plays a crucial role in the creative process and should not be overlooked. Future investigations in the field of creativity assessment may benefit from using a broader range of robust and varied metrics that encompass both convergent and divergent thinking, to yield a more thorough evaluation of creative aptitude. Furthermore, the present study assessed participants’ everyday creativity as little-C which can be found in everyone (Kaufman & Beghetto, 2009), although it should be noted that creativity within organizational contexts is mostly associated with professional creativity (pro-C). Pro-C represents the developmental and effortful progression beyond little-c, and its concept is consistent with the expertise acquisition approach of creativity (Kaufman & Beghetto, 2009). While the alternative uses task (AUT) provides a well-established measure of divergent thinking and creative potential, it does not fully capture domain-specific or applied creativity in workplace settings. Additionally, AUT relies on fluency-based metrics, which may overemphasize idea quantity rather than the feasibility and impact of creative contributions in professional environments. Further research may be warranted to explore in further depth the correlation between distinct subtypes of narcissism and professional creativity on organizational levels. Finally, the division of participants into grandiose, vulnerable, and non-narcissistic groups based on a standard deviation of 1 or −1 lacks a clear rationale. While this approach is commonly used in social sciences to distinguish between psychological traits, there is no universally established cut-off in the narcissism literature for categorizing individuals into these specific subtypes. This classification method, though practical, may introduce variability in group assignment. However, our descriptive statistical analyses confirmed that the grandiose and vulnerable narcissism groups exhibited distinct patterns on their respective B-PNI dimensions (shown as Table 1), providing empirical support for the validity of this grouping approach. Future research may benefit from exploring alternative classification methods, such as latent profile analysis or empirically derived cut-offs from larger normative samples, to enhance the robustness of subgroup distinctions.

## 5. Conclusions

The present study aimed to investigate the relationship between different subtypes of narcissism and creativity, as well as the mediating role of self-esteem in both. Grandiose narcissistic participants were significant reported higher creativity states and higher fluency in creativity performance than vulnerably narcissistic participants. Self-esteem mediated the relationship between grandiosity and vulnerability of narcissism and creativity. By tailoring incentives and task assignments to employees’ narcissistic subtypes, companies can maximize creative potential. Enhancing self-esteem across the board can also help boost overall creativity and productivity, fostering a more innovative and high-performing work environment.

## Figures and Tables

**Table 1:** The average and standard variations of narcissism and creativity (*n* = 110).

	Grandiose Narcissism (*n* = 46)		Vulnerable Narcissism (*n* = 33)		Non-Narcissism (*n* = 31)	
	Mean	*SD*	Mean	*SD*	Mean	*SD*
BNPI—Grandiose	4.5	0.5	3.7	0.3	2.5	0.9
BNPI—Vulnerable	2.6	0.5	4.0	0.4	2.8	0.3
Creativity state—Originality	3.2	1.1	2.8	0.8	2.5	1.1
Creativity state—Fluency	3.5	0.9	2.7	0.7	2.5	1.1
Creativity state—Usefulness	3.7	0.9	2.9	1.1	2.7	1.1
Creativity state—Overall	3.3	1.2	2.6	1.0	2.6	1.1
Creativity performance—Fluency	7.4	4.3	5.6	3.1	5.5	3.5
Creativity performance—Flexibility	5.1	2.1	4.4	1.8	4.2	2.1
Creativity performance—Originality	2.8	0.6	2.6	0.8	2.6	0.7

**Table 2:** Correlations between narcissism, self-esteem and creativity (*N* = 571).

	Mean	*SD*	1	2	3	4	5	6	7	8	9	10
1 GrdNar	3.6	0.7	1									
2 VulNar	2.9	0.7	0.44 **	1								
3 Self-Esteem	2.9	0.4	0.19 **	−0.20 **	1							
4 Creativity state—Originality	3.0	1.0	0.17 **	−0.08	0.35 **	1						
5 Creativity state—Flexibility	3.0	0.9	0.22 **	−0.06	0.35 **	0.75 **	1					
6 Creativity state—Usefulness	3.2	1.0	0.20 **	−0.04	0.34 **	0.67 **	0.72 **	1				
7 Creativity state—Overall	2.9	1.0	0.19 **	−0.05	0.37 **	0.67 **	0.67 **	0.68 **	1			
8 Creativity performance—Fluency	6.2	3.7	0.18 **	−0.07	0.23 **	0.22 **	0.20 **	0.18 **	0.13 **	1		
9 Creativity performance—Flexibility	4.5	2.0	0.18 **	−0.04	0.20 **	0.21 **	0.19 **	0.17 **	0.12 **	93 **	1	
10 Creativity performance—Originality	2.7	0.7	0.15 **	−0.004	0.13 **	0.24 **	0.23 **	0.18 **	0.12 **	0.69 **	0.73 **	1

Note: ** *p* < 0.01. GrdNar refers to grandiose narcissism, VulNar refers to vulnerable narcissism.

**Table 3:** Standardized direct, indirect, and total effects of the hypothesized model.

	Point Estimate	Product of Coefficients		Bootstrapping		Two-Tailed Significance
				Percentile 95% CI		
		*SE*	*Z*	Lower	Upper	
GndNar → Creative states	0.15	0.04	3.64	0.08	0.22	*p* < 0.01
GndNar → Creativity performance	0.20	0.05	4.17	0.12	0.28	*p* < 0.01
GndNar → Self-esteem	0.34	0.04	7.64	0.26	0.41	*p* < 0.01
VulNar → Creative performance	−0.10	0.05	−2.17	−0.18	−0.02	*p* = 0.038
VulNar → Self-esteem	−0.35	0.04	−8.05	−0.42	−0.27	*p* < 0.01
Self-esteem → Creative states	0.38	0.40	0.94	0.31	0.44	*p* < 0.01
Self-esteem → Creativity performance	0.16	0.04	3.77	0.09	23	*p* < 0.01

Note: GndNar refers to grandiose narcissism; VulNar refers to vulnerable narcissism.

## Data Availability

Data are contained within the article.

## References

[B1-behavsci-15-00273] Acar S., Runco M. A. (2019). Divergent thinking: New methods, recent research, and extended theory. Psychology of Aesthetics, Creativity, and the Arts.

[B2-behavsci-15-00273] Ackerman R. A., Donnellan M. B., Wright A. G. (2019). Current conceptualizations of narcissism. Current Opinion in Psychiatry.

[B3-behavsci-15-00273] Ackerman R. A., Witt E. A., Donnellan M. B., Trzesniewski K. H., Robins R. W., Kashy D. A. (2011). What does the narcissistic personality inventory really measure?. Assessment.

[B4-behavsci-15-00273] American Psychiatric Association (2000). Diagnostic and statistical manual of mental disorders.

[B5-behavsci-15-00273] Benedek M., Franz F., Heene M., Neubauer A. C. (2014). Differential effects of cognitive inhibition and intelligence on creativity. Personality and Individual Differences.

[B6-behavsci-15-00273] Bosson J. K., Lakey C. E., Campbell W. K., Zeigler-Hill V., Jordan C. H., Kernis M. H. (2008). Untangling the links between narcissism and self-esteem: A theoretical and empirical review. Social and Personality Psychology Compass.

[B7-behavsci-15-00273] Brummelman E., Thomaes S., Sedikides C. (2016). Separating narcissism from self-esteem. Current Directions in Psychological Science.

[B8-behavsci-15-00273] Cain N. M., Pincus A. L., Ansell E. B. (2008). Narcissism at the crossroads: Phenotypic description of pathological narcissism across clinical theory, social/personality psychology, and psychiatric diagnosis. Clinical Psychology Review.

[B9-behavsci-15-00273] Campbell W. K., Rudich E. A., Sedikides C. (2002). Narcissism, self-esteem, and the positivity of self-views: Two portraits of self-love. Personality and Social Psychology Bulletin.

[B10-behavsci-15-00273] Chang L., Gong Z. (2023). The relationship between narcissism and creativity: A chain/serial mediation model. Personality and Individual Differences.

[B11-behavsci-15-00273] Cohen J. (1988). Statistical power analysis for the behavioral sciences.

[B12-behavsci-15-00273] Deng X. P., Zhang X. K. (2011). Understanding the relationship between self-esteem and creativity: A meta-analysis. Advances in Psychological Science.

[B13-behavsci-15-00273] Dickinson K. A., Pincus A. L. (2003). Interpersonal analysis of grandiose and vulnerable narcissism. Journal of Personality Disorders.

[B14-behavsci-15-00273] Furnham A., Hughes D. J., Marshall E. (2013). Creativity, OCD, narcissism and the big five. Thinking Skills and Creativity.

[B15-behavsci-15-00273] Gebauer J. E., Sedikides C. (2018). Communal narcissism: Theoretical and empirical support. Handbook of trait narcissism: Key advances, research methods, and controversies.

[B16-behavsci-15-00273] Geukes K., Nestler S., Hutteman R., Dufner M., Küfner A. C. P., Egloff B., Denissen J. J. A., Back M. D. (2017). Puffed-up but shaky selves: State self-esteem level and variability in narcissists. Journal of Personality and Social Psychology.

[B17-behavsci-15-00273] Gilhooly K. J., Fioratou E., Anthony S. H., Wynn V. (2007). Divergent thinking: Strategies and executive involvement in generating novel uses for familiar objects. British Journal of Psychology.

[B19-behavsci-15-00273] Goncalo J. A., Flynn F. J., Kim S. H. (2010). Are two narcissists better than one? The link between narcissism, perceived creativity, and creative performance. Personality and Social Psychology Bulletin.

[B18-behavsci-15-00273] Goncalo J. A., Krause V., Miles J. A. (2014). Narcissism and creativity over time: Toward a dynamic model. New directions in management and organization theory.

[B20-behavsci-15-00273] Gregg A. P., Sedikides C. (2010). Narcissistic fragility: Rethinking its links to explicit and implicit self-esteem. Self and Identity.

[B21-behavsci-15-00273] Guilford J. P. (1967). The nature of human intelligence.

[B22-behavsci-15-00273] Han W., Feng X., Zhang M., Peng K., Zhang D. (2019). Mood states and everyday creativity: Employing an experience sampling method and a day reconstruction method. Frontiers in Psychology.

[B23-behavsci-15-00273] Hart W., Tortoriello G. K., Richardson K., Breeden C. J. (2020). Substantive vs. superficial self-enhancement: Differentiating narcissism constructs from self-esteem following failure. Personality and Individual Differences.

[B24-behavsci-15-00273] Jauk E., Sordia N. (2018). On risks and side effects: Does creative accomplishment make us narcissistic? Creativity. Theories–Research-Applications.

[B25-behavsci-15-00273] Ji Y., Liu H., Liu S., Xu M., Lin Z. (2023). Are narcissists more creative? Only if we believe it: How narcissism can relate to creativity. Frontiers in Psychology.

[B26-behavsci-15-00273] Jonason P. K., Abboud R., Tomé J., Dummett M., Hazer A. (2017). The dark triad traits and individual differences in self-reported and other-rated creativity. Personality and Individual Differences.

[B27-behavsci-15-00273] Kaufman J. C., Beghetto R. A. (2009). Beyond big and little: The four c model of creativity. Review of General Psychology.

[B28-behavsci-15-00273] Kemple K. M., David G. M., Wang Y. (1996). Preschoolers’ creativity, shyness, and self-esteem. Creativity Research Journal.

[B29-behavsci-15-00273] Kohut H. (1966). Forms and transformations of narcissism. Journal of the American Psychoanalytic Association.

[B30-behavsci-15-00273] Kris E. (1953). Psychoanalytic explorations in art. Journal of Aesthetics and Art Criticism.

[B31-behavsci-15-00273] Lebuda I., Figura B., Karwowski M. (2021). Creativity and the dark triad: A meta-analysis. Journal of Research in Personality.

[B32-behavsci-15-00273] Maciantowicz O., Zajenkowski M., Thomaes S. (2022). Vulnerable and grandiose narcissism in adolescence: Associations with anger and hostility. Current Psychology.

[B33-behavsci-15-00273] Mann D. (2022). Narcissism and the lure of grandiosity over reality: Reflections on a narcissistic inhibition in creativity. British Journal of Psychotherapy.

[B34-behavsci-15-00273] Mao J., Quan J., Li Y., Xiao J. (2021). The differential implications of employee narcissism for radical versus incremental creativity: A self-affirmation perspective. Journal of Organizational Behavior.

[B35-behavsci-15-00273] Martinsen Ø. L., Arnulf J. K., Furnham A., Lang-Ree O. C. (2019). Narcissism and creativity. Personality and Individual Differences.

[B36-behavsci-15-00273] Miller J. D., Campbell W. K. (2008). Comparing clinical and social-personality conceptualizations of narcissism. Journal of Personality.

[B37-behavsci-15-00273] Miller J. D., Hoffman B. J., Gaughan E. T., Gentile B., Maples J., Keith Campbell W. (2011). Grandiose and vulnerable narcissism: A nomological network analysis. Journal of personality.

[B38-behavsci-15-00273] Miller J. D., Widiger T. A., Campbell W. K. (2010). Narcissistic personality disorder and the DSM-5. Journal of Abnormal Psychology.

[B39-behavsci-15-00273] Morf C. C., Rhodewalt F. (2001). Unraveling the paradoxes of narcissism: A dynamic self-regulatory processing model. Psychological Inquiry.

[B40-behavsci-15-00273] Orth U., Robins R. W., Meier L. L., Conger R. D. (2016). Refining the vulnerability model of low self-esteem and depression: Disentangling the effects of genuine self-esteem and narcissism. Journal of Personality and Social Psychology.

[B41-behavsci-15-00273] Pincus A. L., Jonason P. K. (2023). A brief overview of the pathological narcissism inventory. Shining light on the dark side of personality.

[B42-behavsci-15-00273] Pincus A. L., Ansell E. B., Pimentel C. A., Cain N. M., Wright A. G., Levy K. N. (2009). Initial construction and validation of the pathological narcissism inventory. Psychological Assessment.

[B43-behavsci-15-00273] Raskin R. (1980). Narcissism and creativity: Are they related?. Psychological Reports.

[B44-behavsci-15-00273] Rohmann E., Neumann E., Herner M. J., Bierhoff H. W. (2012). Grandiose and vulnerable narcissism: Self-construal, attachment, and love in romantic relationships. European Psychologist.

[B45-behavsci-15-00273] Rosenberg M. (1965). Society and the adolescent self-image.

[B46-behavsci-15-00273] Runco M. A., Acar S. (2012). Divergent thinking as an indicator of creative potential. Creativity Research Journal.

[B47-behavsci-15-00273] Runco M. A., Jaeger G. J. (2012). The standard definition of creativity. Creativity Research Journal.

[B48-behavsci-15-00273] Samuel D. B., Widiger T. A. (2008). A meta-analytic review of the relationships between the five-factor model and DSM-IV-TR personality disorders: A facet level analysis. Clinical Psychology Review.

[B49-behavsci-15-00273] Schoenleber M., Roche M. J., Wetzel E., Pincus A. L., Roberts B. W. (2015). Development of a brief version of the pathological narcissism inventory. Psychological Assessment.

[B50-behavsci-15-00273] Sedikides C. (2021). In search of narcissus. Trends in Cognitive Sciences.

[B51-behavsci-15-00273] Sedikides C., Gregg A. P. (2008). Self-enhancement: Food for thought. Perspectives on Psychological Science.

[B52-behavsci-15-00273] Sedikides C., Rudich E. A., Gregg A. P., Kumashiro M., Rusbult C. (2004). Are normal narcissists psychologically healthy?: Self-esteem matters. Journal of Personality and Social Psychology.

[B53-behavsci-15-00273] Silvia P. J., Phillips A. G. (2004). Self-awareness, self-evaluation, and creativity. Personality and Social Psychology Bulletin.

[B54-behavsci-15-00273] Sternberg R. J., Karami S. (2021). An 8p theoretical framework for understanding creativity and theories of creativity. The Journal of Creative Behavior.

[B55-behavsci-15-00273] Tromp C., Sternberg R. J. (2022). Dynamic creativity: A person × task × situation interaction framework. The Journal of Creative Behavior.

[B56-behavsci-15-00273] Weiss B., Miller J. D., Foster J. D., Brunell A., Hermann T. (2018). Distinguishing between grandiose narcissism, vulnerable narcissism, and narcissistic personality disorder. The handbook of trait narcissism: Key advances, research methods, and controversies.

[B57-behavsci-15-00273] Wink P. (1991). Two faces of narcissism. Journal of Personality and Social Psychology.

[B58-behavsci-15-00273] Zeigler-Hill V., Besser A. (2013). A glimpse behind the mask: Facets of narcissism and feelings of self-worth. Journal of Personality Assessment.

[B59-behavsci-15-00273] Zhang W., Sjoerds Z., Hommel B. (2020). Metacontrol of human creativity: The neurocognitive mechanisms of convergent and divergent thinking. NeuroImage.

